# A Large Measurable Range Capacitance-to-Digital Converter for Smart Humidity Sensors

**DOI:** 10.3390/mi10090561

**Published:** 2019-08-24

**Authors:** Rongshan Wei, Weiwen Lin, Xiaoxia Xiao, Qunchao Chen, Fanyang Li

**Affiliations:** College of Physics and Information Engineering, Fuzhou University, Fuzhou 350116, China

**Keywords:** humidity sensor, sigma-delta modulator, cascade of integrators with a feed-forward (CIFF)

## Abstract

This study aims to propose a capacitance-to-digital converter (CDC) based on a third-order cascade of integrators with a feed-forward (CIFF) incremental sigma-delta modulator for smart humidity sensor application. Disguised zoom-in technology was proposed to enlarge the measurable range of the CDC. The input range of the CDC was 0–388 pF. The proposed CDC was realized using 0.18 μm complementary metal-oxide-semiconductor technology. Results show that the CDC performs a 13-bit capacitance-to-digital conversion in 0.8 ms. The analog system consumes 169.7 μA from a 1.8 V supply, which corresponds to a figure of merit (FOM) of 3.0 nJ/step. The proposed CDC was combined with a HS1101 humidity sensor to demonstrate its incorporation in an overall system design. The resolution was 0.7% relative humidity (RH) over a range of 30%–90% RH.

## 1. Introduction

Relative humidity is an important factor in industrial processes, human health, and preservation of perishable food and cultural relics [1,2]. It is used to characterize the ratio (in percentage) of the humidity in the air to the saturated absolute humidity at the same temperature and pressure.

A low-cost thermal-conductivity-based humidity sensor has been previously reported [3], having a simple implementation principle but high power consumption. A fully integrated humidity sensor for radio frequency identification (RFID) applications [4], although consuming a small current took considerable time to take the measurement. A complementary metal-oxide-semiconductor (CMOS) humidity sensor with a short measurement time [5] had a limited measurable range of the capacitance-to-digital converter (CDC) of only 0–1.76 pF.

A capacitance-to-voltage front-end CDC has been proposed [6] which relies on a successive approximation register analog-to-digital converter (ADC) such that the effective number of bits (ENOB) is only 8.35 bits. Another study [7] presented a capacitance-to-voltage converter using capacitive microelectromechanical systems (MEMS) that included a dual-slope ADC, significantly increasing the conversion time to 20 ms. 

A time-based CDC that performs capacitance-to-time conversion followed by time-to-digital conversion has also been reported [8]. However, aside from requiring a cumbersome working process, the signal-to-noise ratio (SNR) of this device is as low as 45.14 dB owing to error accumulation in the two conversion steps. Another proposed CDC [9] can operate in either fast or slow modes; however, it uses a charge amplifier that introduces a significant amount of additional error and consumes a large amount of power (as much as 1 W). A high-resolution CDC was recently developed [10], but its input capacitance range is only −4–4 pF, and it has a long measurement time of up to 62 ms.

However, these sensors did not meet the market’s requirements of low power consumption, large capacitance input range, and fast measurements.

Herein, a novel CDC is proposed to address the abovementioned issues. A new method called disguised zoom-in technology was employed to enlarge the measurable range to 0–388 pF. Low power consumption and a short conversion time were also focused on in the design. The proposed CDC’s total power consumption was 305.46 uW, and the measurements only took 0.8 ms. The measurable relative humidity range was 30%–90%.

The proposed CDC combined with a humidity sensor system could be applied to the industrial production of a relative humidity controller and to monitor humidity in daily life.

This article is organized as follows. Section 2 describes the principle and operation of the CDC. Section 3 discusses the overall structure of the CDC. The ADC and the concept of disguised zoom-in technology is also presented in detail in this section. Section 4 presents the results recorded by the proposed CDC and compares them with the data obtained from previously reported CDCs. Finally, some conclusions are presented in Section 5.

## 2. CDC Operation Principle

**Figure 1:**
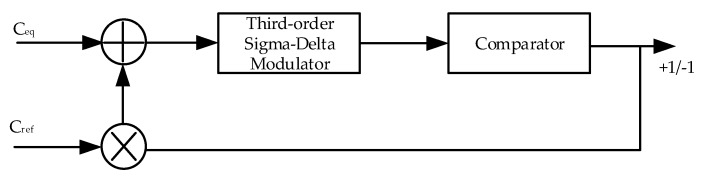
Charge balanced operation of the capacitance-to-digital converter (CDC).

The CDC structure is essentially a charge balance that uses a sigma-delta modulator. A third-order modulator, as shown in Figure 1, illustrates the operation of the CDC, where C_eq_ represents the capacitance to be measured and C_ref_ denotes the reference capacitance. 

Let us assume that the modulator has completed the reset operation; thus, it is ready to perform a new round of measurements. During each conversion cycle, V_DD_ charges C_eq_ first. It is determined by the previous bitstream such that V_ref_ or −V_ref_ charges C_ref_. When the previous bitstream is positive, −C_ref_V_ref_ is added to C_eq_V_DD_. Otherwise, C_ref_V_ref_ is added to C_eq_V_DD_. The two parts of the charge are added to each other and transmitted to an integrator, the output of which flows to the comparator, generating a second bitstream. The polarity of the first bitstream after the reset does not impact the final result substantially.

The integrators of the modulator are reset before each measurement to prevent interference from any residual charge from the previous conversion [11,12]. 

The entire charge balancing process can be represented by Equation (1), where μ is expressed by Equation (2).

(1) $$V_{DD}\cdot C_{\mathrm{eq}}-\mu \cdot V_{\mathrm{ref}}\cdot C_{\mathrm{ref}}+(1-\mu )\cdot V_{\mathrm{ref}}C_{\mathrm{ref}}=0$$

(2) $$\mu =\frac{C_{\mathrm{eq}}}{2C_{\mathrm{ref}}}+\frac{1}{2}$$

According to the above description of CDC operation, the CDC first converts the capacitance into the charge via charging C_eq_. Then, CDC superimposes the charge that is controlled by the weight of the bitstream to balance it. The residual charge is sent to the modulator until it is finally balanced.

The CDC uses the ratiometric measurement principle for ADC conversion. The density of the bitstream is the ratio of C_eq_ to C_ref_. Thus, C_eq_ can be calculated, as long as the density of the bitstream is processed, according to Equation (2).

## 3. Structure Implementation

**Figure 2:**
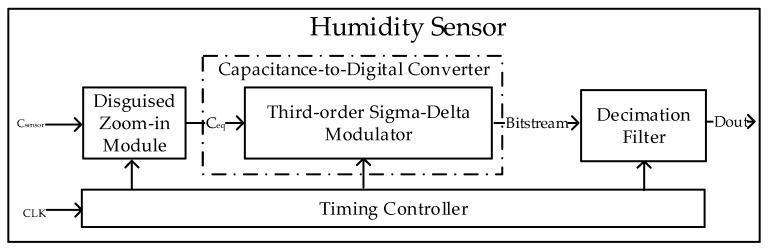
Overview of the analog-to-digital converter (ADC).

Figure 2 shows the ADC used herein. The humidity sensor mainly comprises three parts. The disguised zoom-in module was used to scale the humidity capacitance, eliminate the baseline capacitance, and amplify the input dynamic capacitance range of the CDC. The sigma-delta modulator and decimation filter form a complete ADC. The timing controller is mainly used for the control and timing coordination of the entire circuit.

The output bitstream was saved in the form of a text document using the Data Acquisition (DAQ) Assistant and then processed in MATLAB R2013b.

The disguised zoom-in module and sigma-delta modulator are described in detail below.

### 3.1. Disguised Zoom-In Technology

Humidity capacitance sensors were implemented off-chip because of process limitations. CDC was implemented on-chip. The HS1101 relative humidity sensor was adopted as the humidity capacitor. The humidity sensitive capacitor can operate between −40 °C and 125 °C, and the range of relative humidity operation is 0%–100%. The polynomial response is presented by Equation (3) [13]. 

(3) $$C(pF)=180\cdot (1.25\cdot 10^{-7}RH^{3}-1.36\cdot 10^{-5}\cdot RH^{2}+2.19\cdot 10^{-3}RH+0.9)$$

The humidity sensitive capacitor has a capacitance of 162–199.44 pF at a relative humidity of 0%–100% at 25 °C. As the dynamic capacitance of the sensor is 37.44 pF and the baseline capacitance is as large as 162 pF, it exceeds the measurement range of most CDCs reported. Therefore, to enable normal measurement, the disguised zoom-in technique was proposed.

The disguised zoom-in technology contains two important processes.

**Figure 3:**
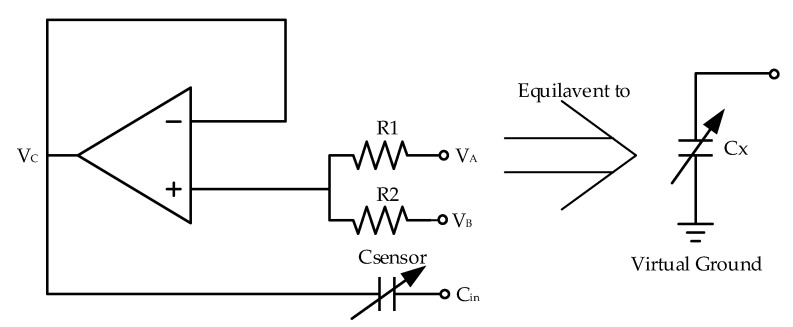
Scalable C_x_ circuit.

The baseline capacitance and dynamic capacitance are both beyond the CDC measurable range; therefore, the humidity sensitive capacitor must first be scaled, which is the first step in disguised zoom-in technology. This process is shown in Figure 3, where C_sensor_ denotes the humidity sensor, and R_1_ and R_2_ are resistors for voltage proportional amplification. C_in_ is the port where the CDC accesses the capacitance to be tested. V_A_ and V_B_ are used to charge the capacitance to be measured for the subsequent charge balancing of the CDC. The output voltage of the op amp is V_C_.

Equation (4) can be simply obtained by including the character of a virtual short in the op amp. Note that V_B_ is the inverse signal of V_A_, and β denotes the amplification factor of the capacitance, which is also a voltage amplification factor. Equation (5) can thus be obtained from Equation (4) as follows.

(4) $$\frac{V_{A}-V_{C}}{R_{1}}+\frac{V_{B}-V_{C}}{R_{2}}=0$$

(5) $$\beta =\frac{V_{A}}{V_{C}}=\frac{R_{2}+R_{1}}{R_{2}-R_{1}}$$

Equation (6) can be obtained using the principle of conservation of charge. Therefore, the equivalent capacitance can also be expressed by Equation (7), where C_x_ represents the equivalent capacitance after being scaled.

(6) $$Q_{sensor}=(\frac{1}{\beta }C_{sensor})\cdot (\beta V_{C})$$

(7) $$C_{x}=\frac{C_{sensor}}{\beta }$$

**Figure 4:**
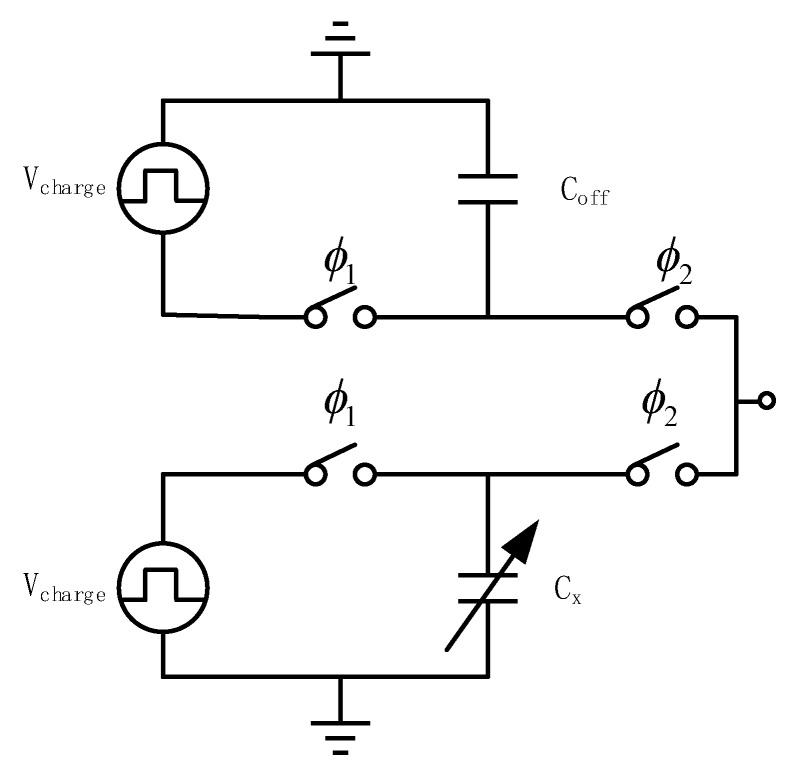
Zoom-in circuit.

The zoom-in technology connects the capacitor to be tested, C_x_, in parallel with the compensation capacitor, C_off_. Using zoom-in technology, the adverse effects of baseline capacitance can be eliminated. This process is shown in Figure 4.

During the integrating phase, $\phi_{1}$, the capacitance to be measured and compensation capacitor are charged by two equal-amplitude inverted excitation voltages, V_charge_. During the sample phase, $\phi_{2}$, the capacitance to be tested, C_x_, and the compensation capacitor, C_off_, are connected in parallel to the interface. Equation (8) is obtained using the principle of conservation of charge, wherein the equivalent capacitance to CDC is given by C_x_ − C_off_. C_eq_ represents the final equivalent capacitance in the disguised zoom-in technology and the result is shown in Equation (9).

(8) $$Q_{eq}=V_{charge}C_{x}-V_{charge}C_{off}$$

(9) $$C_{eq}=C_{x}-C_{off}=\frac{C_{sensor}}{\beta }-C_{off}$$

The compensation capacitor, C_off_, is controlled by a binary toggle switch, where the toggle has been designed off-chip. Through the four toggle switches of S0000–S1111, C_off_ can be controlled between 0–7.5 pF using individual steps of 0.5 pF.

The baseline capacitance of the humidity sensitive capacitor is eliminated in the second process of the disguised zoom-in technique. In addition, the scaling process of the disguised zoom-in technique further scales the dynamic capacitance of the humidity sensitive capacitor such that the entire dynamic range falls within the input range of the CDC.

### 3.2. Sigma-Delta Converter Structure

As the capacitance of the humidity sensor changes relatively slowly with ambient humidity and the capacitance is converted to the form of a charge input, the input amount is approximately direct current (DC) voltage for the ADC. Therefore, an incremental sigma-delta ADC with a third-order cascade of integrators and a feed-forward structure was selected using a one-bit quantizer [14,15].

**Figure 5:**
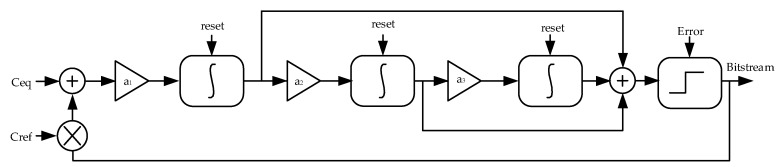
Third-order cascade of integrators with a feed-forward (CIFF) modulator structure and the main signal flow.

In Figure 5, C_eq_ represents the humidity sensor capacitance after application of the disguised zoom-in technology. C_ref_ denotes a reference capacitor used in the design. Some quantization errors are generated during the quantization process; these are marked as “Error” in Figure 5.

The signal transfer function and noise transfer function are denoted as STF(z) and NTF(z), respectively. Equations (10) and (11) can be easily obtained by inspecting Figure 5.

(10) $$STF(z)=\frac{a_{1}z^{-1}+(a_{1}a_{2}-2a_{1})z^{-2}+(a_{1}-a_{1}a_{2}+a_{1}a_{2}a_{3})z^{-3}}{{\left(1-z^{-1}\right)}^{-3}}$$

(11) $$NTF(z)=\frac{1}{1-\frac{a_{1}z^{-1}+(a_{1}a_{2}-2a_{1})z^{-2}+(a_{1}-a_{1}a_{2}+a_{1}a_{2}a_{3})z^{-3}}{{\left(1-z^{-1}\right)}^{-3}}}$$

Usually, the zeros of the noise transfer function are concentrated at z = 1. If the zeros of the noise transfer function are evenly distributed in the signal baseband by some mathematical software, a better attenuation effect on the quantization noise can be achieved [16,17]. With the help of Simulink and the DS toolbox in MATLAB, zero optimization of the third-order CIFF can be performed. The final values of a_1_, a_2_, and a_3_ are 0.16, 0.5, and 0.2, respectively.

### 3.3. Circuits Implementation

To achieve a higher resolution in the case of the ADC, the architecture and its corresponding coefficients need to carefully be considered and the specific circuits that constitute the internal structure must carefully be designed. A good circuit structure can improve accuracy, reduce power, and increase speed. Therefore, the circuits that constitute the ADC are discussed in detail in this section.

**Figure 6:**
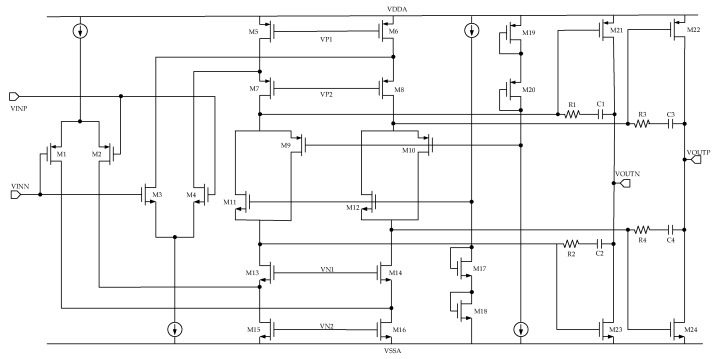
First-stage integrator op amp.

The first-stage op amp that constitutes the CDC must withstand operation at very high or low voltages, which are caused by the initial input voltage when the capacitance to be tested is large or the charge-balancing circuit starts to work and the feedback negative voltage is too large. This results in a higher or lower input voltage of the first-stage integrator. Therefore, we use rail-to-rail inputs in our design to accommodate such harsh conditions. For the output of the first-stage op amp, a class AB structure is adopted, which can improve the current-utilization efficiency. The specific circuit of the first-stage op amp is shown in Figure 6.

**Figure 7:**
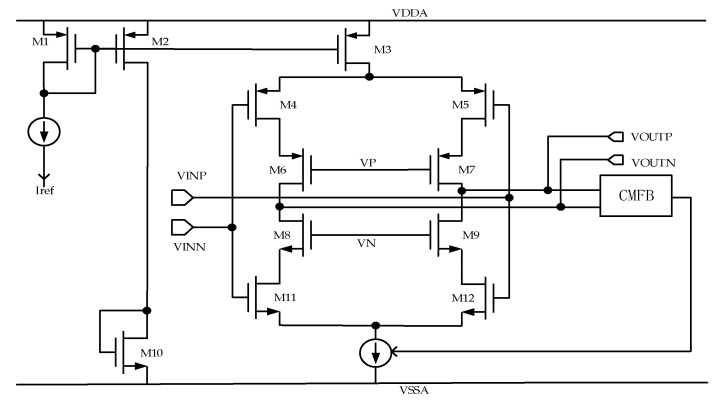
Second- and third-stage integrator op amps.

The performance requirements of the second- and third-stage op amps are not as high as those of the first-stage system, so the power consumption is reduced under the premise of satisfying the basic performance (medium gain and transconductance). A current-starved op amp, which greatly reduces power consumption, is used [18]. The corresponding circuit is shown in Figure 7. The CMFB is a common-mode feedback of the differential op amp that stabilizes the common-mode voltage at the output.

The first-, second-, and third-stage op amps consume 161, 0.8, and 0.4 µA. The reason why the first-stage op amp consumes the most current is that a larger bandwidth and higher transconductance are required to quickly establish the integrator signal. The second- and third-stage op amps consume less current because the requirements for the op amp are relaxed and the current-starved op amp improves the current utilization efficiency.

**Figure 8:**
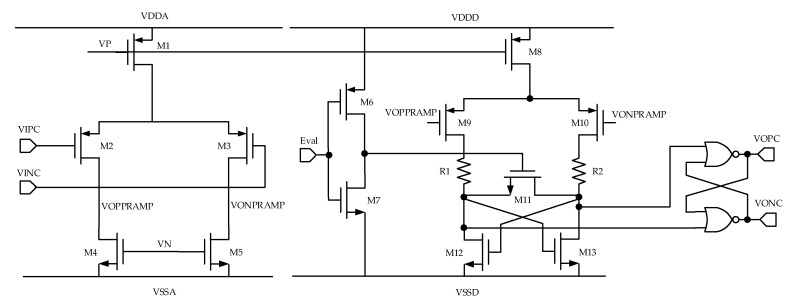
The circuit of the comparator.

For the comparator design, a combination of a preamplifier and static latch is used. The preamplifier can pre-amplify the signal to be compared, thus increasing the speed of comparison. The use of preamplifiers with latches prevents kickback noise [19]. Using a static latch allows the comparator to operate at a higher speed. The comparator is shown in Figure 8. “Eval” represents the valid signal. When “Eval” is high, it compares. When it is low, it does not compare, but the signal is latched.

## 4. Measurement Results

**Figure 9:**
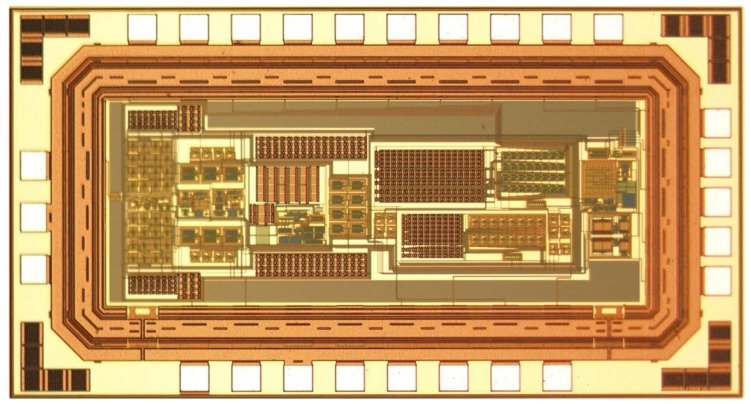
Micrograph of the CDC chip.

The proposed CDC for a smart humidity sensor was implemented in 0.18 µm CMOS technology provided by Semiconductor Manufacturing International Corporation (SMIC). Figure 9 shows the electron micrograph of the CDC chip. The die area is 0.57 mm^2^. To flexibly handle and reduce the area occupied by the digital circuit, the decimation filter was implemented off-chip.

The CDC is powered by a 1.8 V supply voltage with a sampling frequency of 250 kHz. The analog circuit consumes 169.7 µA of current. The measurement time of the CDC is 0.8 ms. The resolution of the CDC is 13 bits. Furthermore, the figure of merit (FOM), which is defined in Equation (12), is 3.0 nJ/step. In similar studies [20,21], the FOMs were 5.2 and 6.7 nJ/step, respectively. Hence, the approach proposed in this study offers a slight advantage of FOM than the previous studies.

(12) $$FOM=\frac{Power_{total}\times T_{measurement}}{2^{ENOB}}$$

**Figure 10:**
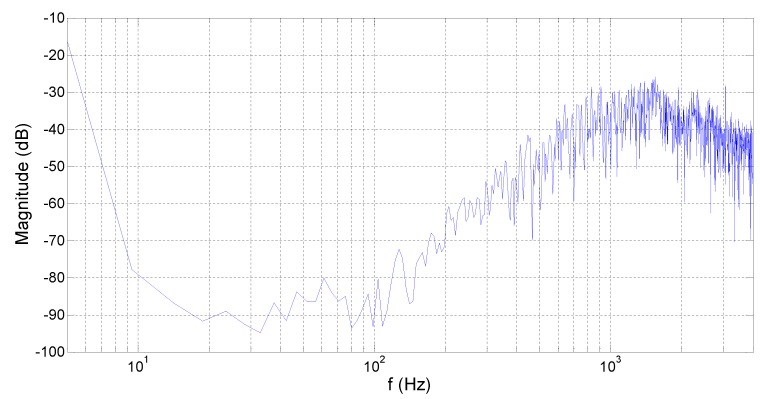
Output spectrum of the bitstream.

Fast Fourier transform was performed on the bitstream acquired in the text. The output spectrum is shown in Figure 10. The noise in the spectrum indicates that the chip achieved third-order noise shaping and the noise floor reached −88 dB. 

By controlling the capacitance of C_off_, the input range could be adjusted. Using the proposed disguised zoom-in technology the whole input range was enlarged to 0–388 pF.

The packaged chips were placed in a climate chamber for testing to quantitatively analyze the performance of the design. Owing to the deviation in the foundry process, a slight deviation is still observed in chips from the same batch. Calibration through the digital back-end can reduce the influence of the deviation to some extent [22]. 

**Figure 11:**
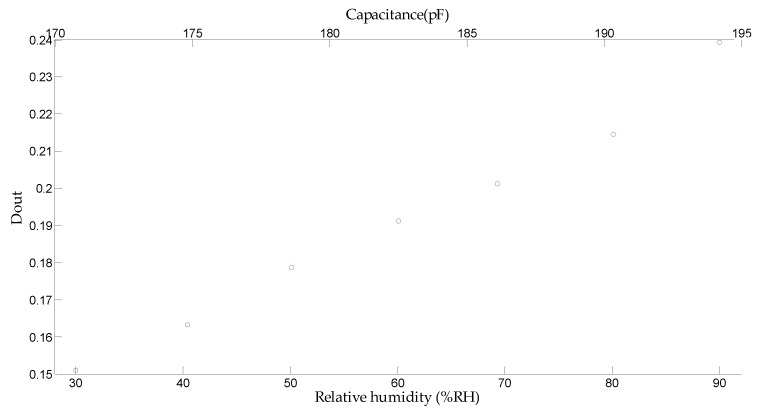
Measured Dout.

Under certain relative humidity, the capacitance can be obtained using Equation (3). The relationship between Dout and capacitance as long as relative humidity is shown in Figure 11, which is measured at 25 °C.

**Figure 12:**
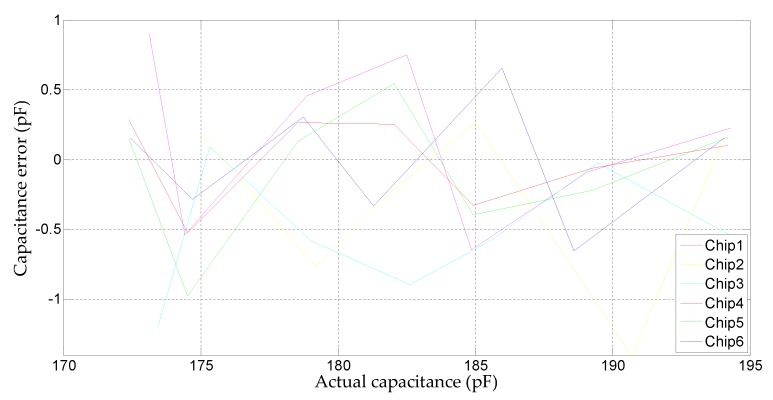
Error of measured capacitance at 25 °C.

The accuracy of the capacitance measured by the CDC will affect the accuracy of the relative humidity after conversion. Figure 12 shows the capacitance error of the measured capacitance corresponding to the relative humidity range of 30%–90% relative humidity (RH).

**Figure 13:**
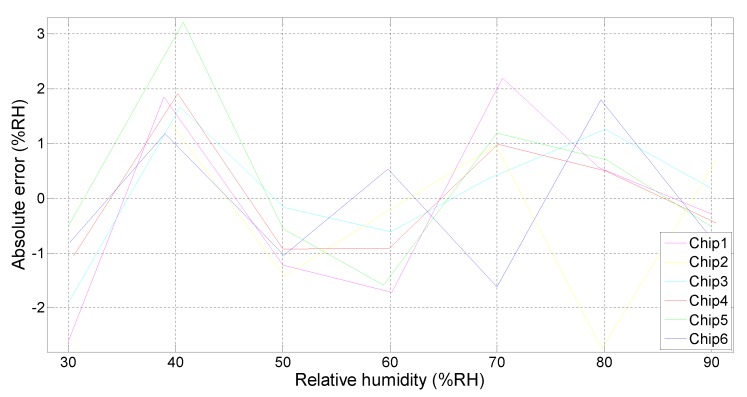
Measured relative humidity error at 25 °C.

The comparison between the measured and actual relative humidity has been done. Figure 13 shows the relative error of the measured relative humidity. Post fitting, the relative humidity error was mostly within 2%, and in extreme cases, it ranged between +3.2% and −2.7%.

Table 1 provides a comparison of the CDC performance with earlier works [23,24,25]. The input range of the capacitance was larger in the CDC presented herein and the power consumption was lower than that in a previous report [24]. The ENOB of this work was also an improvement on an earlier report [23].

As listed in Table 2, compared with earlier humidity sensors [4,26,27], the conversion time of the current humidity sensor was shorter. Furthermore, the supply voltage was lower and had comparable resolution to a previous report [4]. The performance in terms of the relative humidity error was close to that of a previous design [4]. Based on these findings, it was concluded that the developed chip is suitable for fast low-voltage measurements. 

## 5. Conclusions

Herein, a CDC for a humidity sensor was proposed, wherein the use of disguised zoom-in technology enlarged the measurable range of the CDC. The error in the relative humidity was also minimized to a value between +3.2% and −2.7%. Thus, the proposed CDC combined with a humidity sensor system is applicable to a wide range of humidity monitoring applications.

## Figures and Tables

**Table 1:** Performance comparison with previously reported capacitance-to-digital converters (CDCs).

Ref.	Supply Voltage (V)	Power (μW)	Measurement Time (ms)	ENOB (bit)	Input Range (pF)	Accuracy (pF)	FOM (nJ/step)	Area (mm^2^)
[23]	1.2	6.4	0.016	11.6	0–12.66	-	0.000033	0.2
[24]	3.3	760	10.5	16.7	6–22	0.2%	0.074	-
[25]	1.5	52.5	3.2	13	0.27–0.9	-	0.021	0.148
This work	1.8	305.46	0.8	13	0–388	1.1%	3	0.57

ENOB—effective number of bits; FOM—figure of merit.

**Table 2:** Performance comparison with previously reported humidity sensors.

Ref.	Supply Voltage (V)	Power (μW)	Measurement Time (ms)	Relative Humidity Range	Resolution (%RH)	Error (%RH)	Area (mm^2^)
[26]	-	150	1000	20%–80%	0.02	-	-
[4]	3	3.2	1000	0%–100%	0.7	±2.0	-
[27]	1.8	10.5	10.2	20%–90%	0.1	-	0.25
This work	1.8	305.46	0.8	30%–90%	0.7	+3.2/−2.7	0.57
